# Factors Associated with Inadequate Tissue Yield in EUS-FNA for Gastric SMT

**DOI:** 10.5402/2011/619128

**Published:** 2011-06-01

**Authors:** Takuto Suzuki, Makoto Arai, Tomoaki Matsumura, Eiji Arai, Sachio Hata, Daisuke Maruoka, Takeshi Tanaka, Shingo Nakamoto, Fumio Imazeki, Osamu Yokosuka

**Affiliations:** Department of Medicine and Clinical Oncology, Graduate School of Medicine, Chiba University, Inohana 1-8-1, Chiba 260-8670, Japan

## Abstract

*Aims*. Our aim was to identify the factors that made the specimens inadequate and nondiagnostic in endoscopic ultrasound-guided fine-needle aspiration (EUS-FNA) biopsy of suspected submucosal tumors (SMTs). *Methods*. From August 2001 to October 2009, 47 consecutive patients with subepithelial hypoechoic tumors originating in the fourth sonographic layer of the gastric wall suspected as GIST by standard EUS in Chiba University hospital underwent EUS-FNA for histologic diagnosis. We evaluated patient age, sex, location of lesion, size, pattern of growth in a stomach, and pattern of echography retrospectively. We defined a case of gaining no material or an insufficient material for immunohistological diagnosis as nondiagnostic. *Results*. The diagnostic yield of EUS-FNA for the diagnosis of gastric SMTs was 74.5%. Multivariate logistic regression analysis identified that age of under 60 years (compared with patients older than 60 years: odds ratio [OR] = 11.91, 95% confidence interval [CI] = 1.761–80.48) and location of SMT at lower third area (compared with upper or middle third area: OR = 10.62, 95% CI = 1.290–87.42) were the predictive factors for inadequate tissue yield in EUS-FNA. *Conclusions*. The factors associated with inadequate tissue yield in EUS-FNA were younger age and the location of lesion at lower third area in stomach.

## 1. Introduction

Gastrointestinal stromal tumors (GISTs) are the most common mesenchymal tumors of the gastrointestinal (GI) tract. GISTs are frequently discovered by chance on endoscopy performed and are characterized by a bulging of the GI wall with normal, overlying mucosa. GISTs were described in 1983 as tumors in the GI tract and mesentery, characterized by a specific histological and immunohistochemical pattern [1]. Since GIST is now considered as potentially malignant, all GISTs may need to be resected, even small lesions [2]. Differentiating these lesions from benign submucosal lesions such as leiomyomas or schwannomas is crucial. But standard endoscopic biopsy specimens are usually nondiagnostic because the mucosa overlying the submucosal lesion such as GIST is normal.

Endoscopic ultrasonography (EUS), enabling intramural scanning of the GI tract, has been reported to be useful in the diagnosis of submucosal tumor (SMT) and in differentiating SMT from extraluminal lesions [3–5]. EUS is not only capable of characterizing lesions of the GI tract and adjacent structures, but it is also capable of guiding the fine-needle aspiration under real-time ultrasound using a through-the-scope needle aspiration system [6, 7].

Endoscopic ultrasound-guided fine-needle aspiration biopsy (EUS-FNA) is considered to be a reliable and accurate method for the evaluation of submucosal lesions in the GI tract. With this method, it has been reported that the accuracy in gastrointestinal diseases such as SMT was high (38–100%), compared to conventional EUS and that complications including bleeding and infection were rare (0–2.6%) [8, 9].

Therefore, SMT is a suitable target of EUS-FNA. EUS-FNA has been well documented as providing cytologic material for the diagnosis of malignancy, but large studies assessing its utility have mainly looked at its use in sampling lymph nodes, pancreas, and extraintestinal masses [10–16]. Although studies to date evaluating the diagnostic yield of EUS-FNA for the diagnosis of GISTs have been reported, those studies analyzed a small number of patients or discussed about general GI tract other than stomach [17–25]. In addition, there were few studies about factors associated with inadequate tissue yield.

The aim of this study was to determine the diagnostic yield of EUS-FNA of suspected GISTs in the stomach and the factors that made specimens inadequate and nondiagnostic.

## 2. Patients and Methods

### 2.1. Patients

When we could not obtain tissue from SMTs by the usual endoscopic biopsy, we conducted EUS-FNA. From August 2001 to October 2009, 47 consecutive patients with submucosal hypoechoic tumors originating in the fourth sonographic layer of the gastric wall suspected as GIST by standard EUS underwent EUS-FNA for histologic diagnosis at Chiba University Hospital. This study was carried out only at one institute, the Chiba University Hospital, and was approved by the committee of Chiba University ethical. There were 24 males and 23 females, and the mean age was 60.4 years (range 39–81 years).

### 2.2. EUS-FNA

Standard EUS was performed using 12 or 15 MHz ultrasound catheter probe SP-701 (FUJIFILM, Japan). The cases diagnosed as lipoma or cyst by EUS were excluded. EUS-FNA was performed on outpatients having SMT-suspected GIST (>2 cm or if <2 cm, increasing in size), with the patient under conscious sedation (Flunitrazepam, 0.4–1.0 mg i.v.), using a conventional convex scanner echoendoscope. This procedure was performed by three endosonographers. The echoendoscope was connected to an ultrasound scanner ProSound SSD-4000 (ALOKA, Japan). The equipment used consisted of GF-UC240P scope (Olympus, Japan) and 22G NA-200H aspiration needles (Olympus, Japan) or EchoTip Ultrasound needles (Wilson-Cook, USA). Color flow and the Doppler sonography were performed to exclude intervening vascular structures and to select a vessel-free needle track to avoid puncturing vessels. After puncture, the inner needle was pulled out. With the vacuum pressure maintained using the connected 20 mL syringe, the aspiration needle was moved within each tumor in various directions more than 10 times. The saline-containing aspirated material was transferred to a Petri dish, and examined macroscopically.

### 2.3. Immunohistochemistry

Immunoperoxidase stains were subsequently performed on the cell block and representative histologic sections of the tumor using commercially available antibodies against c-kit (CD117), CD34, S-100, and smooth-muscle actin. Diagnosis of GIST was made when pathologic examination showed spindle or epithelioid cells that stained positive for c-kit.

### 2.4. Factors Associated with Inadequate Tissue in EUS-FNA

We defined a case of gaining a sufficient material for immunohistological diagnosis as diagnostic and calculated a diagnostic yield. We divided patients into two groups, diagnostic and nondiagnostic group and evaluated patient age, sex, location of lesion, size long axis (in millimeters), pattern of growth in stomach, and pattern of echography. As for the gastric neoplasm group, the location of the lesion in stomach was classified into three areas: upper third (U), middle third (M), and lower third (L).

### 2.5. Ethics

This study was carried out only at one institute, Chiba University Hospital and was approved by the committee of Chiba University ethical. Written informed consent was obtained from all the patients in accordance with the Declaration of Helsinki.

### 2.6. Statistical Analysis

Data are shown as the mean ± SD. The difference between two groups was tested by the Student's *t*-test. Comparisons of proportions were performed using the chi-square test. *P* < .05 was considered statistically significant. To evaluate the clinical parameters for inadequate tissue yield in EUS-FNA for gastric SMT, logistic regression analysis was performed. SPSS software version 16.0 (SPSS Inc., Chicago, Ill, USA) was used for statistical analysis. 

## 3. Results

### 3.1. Patients and the Results of EUS-FNA

**Figure 1:**
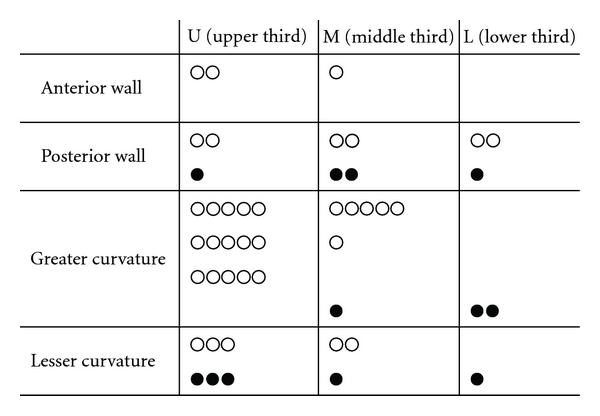
Lesions in the stomach of gastric SMT. Open circles represent diagnostic cases and closed circles represent nondiagnostic cases by EUS-FNA.

**Figure 2:**
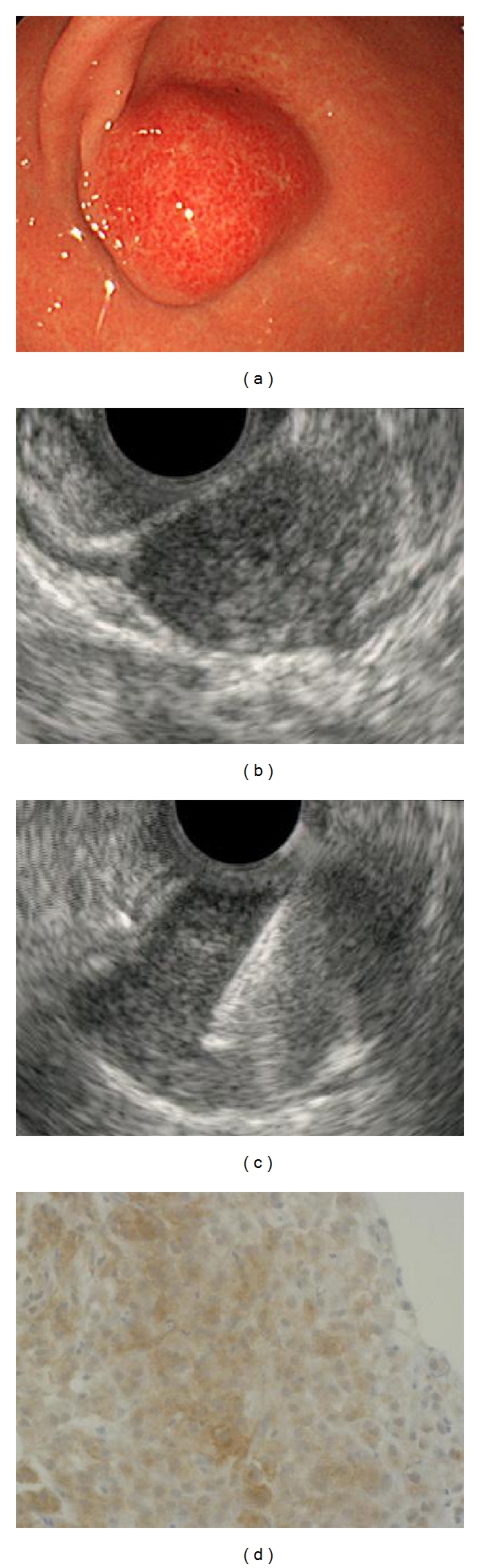
EUS-FNA procedure. Case: 62 years old, female, tumor size 20 mm, area M in stomach. (a) Endoscopy showing submucosal lesion in the stomach, (b) EUS showing submucosal hypoechoic tumor with continuity to the proper muscle layer, (c) the hypoechoic mass shown on EUS was punctured under real-time EUS guidance (EUS-FNA), and (d) cell block specimen from a GIST revealing brown staining, positive for c-kit immunoperoxidase stain.

A total of 47 patients who had undergone EUS-FNA of gastric fourth layer subepithelial lesions were identified. The mean age of patients was 60.4. Of the total of 47 cases, 35 (74.5%) had adequate FNA materials for cytological and histological examination. And 12 (25.5%) were judged as inadequate. The diagnostic yield was 74.5%.  Figure 1 shows the location in the stomach (anterior wall, posterior wall, greater curvature, and lesser curvature) of each case (Figure 1). Final diagnoses after EUS-FNA were GIST (*n* = 27), leiomyoma (*n* = 2), schwannoma (*n* = 1), aberrant pancreas (*n* = 2), malignant lymphoma (*n* = 1), adenocarcinoma (*n* = 1), inflammatory granuloma (*n* = 1), and nondiagnostic (*n* = 12 ). Surgical resection in our hospital was performed in 13 of 27 patients that were diagnosed having GIST in EUS-FNA. To compare the pathologic findings in the tissues from EUS-FNA with the specimen obtained from surgical resection, the accuracy was 100%. No complications occurred (Table 1). EUS-FNA with immunohistochemical analysis was a safe and accurate method in the pretherapeutic diagnosis of SMT. A case with adequate specimen obtained from EUS-FNA was shown in Figure 2.

### 3.2. Factors Associated with Inadequate Tissue Yield

There were 35 patients with diagnostic FNA cytologic findings and on the other hand, 12 patients without diagnostic cytologic findings. The clinical background of the patients was shown in Table 2. There was no statistical significance in sex, size, pattern of growth in a stomach, and pattern of echography in the patients with and without diagnostic cytological finding. On the other hand, the diagnostic yield of EUS-FNA cytology for the diagnosis of SMT was influenced by age and location. Patients were significantly younger in the nondiagnostic group, with a mean age of 49.0 compared with 64.4. The lesions significantly located in L area more than U or M area in the nondiagnostic group (Table 2). The thresholds of age and size of SMT were the average value in the patients. Univariate analysis identified that age of under 60 years and location of SMT at L area were independent factors for inadequate tissue yield in EUS-FNA. Multivariate logistic regression analysis identified that age of under 60 years (compared with patients older than 60 years: odds ratio (OR) = 11.91, 95% confidence interval (CI) = 1.761–80.48) and location of SMT at L area (compared with U or M area: OR = 10.62, 95% CI = 1.290–87.42) were the predictive factors for inadequate tissue yield in EUS-FNA (Table 3).

## 4. Discussion

GISTs are the most commonly identified intramural, submucosal mass in the upper GI tract. These masses are frequently found on endoscopy performed for other reasons, but patients may also present with abdominal pain, bleeding, or symptoms of mass effect [25]. It is well recognized that all GISTs have some degree of malignant potential [1, 2]. Even small localized GISTs may demonstrate malignant features on histologic examination or biologic behavior. However, the mucosal surface of SMT is usually normal, and the biopsy examination by conventional forceps at endoscopy was frequently negative, which showed the difficulty in achieving the material of SMT.

EUS is effective for a diagnosis of these lesions. Lipomas, cysts and submucosal varices have typical features that allow accurate diagnosis based solely on the data gathered from endoscopy and EUS imaging [3, 4, 19]. Additionally, EUS-FNA is more effective for a diagnosis of SMT. In the study of 23 patients with GISTs, it is reported that EUS features alone had a diagnostic accuracy of 77% versus an accuracy of 91% obtained from immunohistochemical analysis from a FNA specimen [18].

The majority of reports on EUS-FNA have focused on pancreatic lesions and lymphadenopathy. Few reports have specifically investigated the use of EUS-FNA in evaluating intramural and extramural structures of the GI tract [24]. Ando et al. retrospectively examined 49 patients with submucosal tumors originating from the fourth sonographic layer. In 4 patients, specimens were inadequate for histopathologic diagnosis, giving a diagnostic yield of 91.8% (45/49) [18]. Okubo et al. examined 18 patients with GIST undergoing both EUS-FNA and surgical resection and calculated a diagnostic yield and sensitivity of 78.0% (14/18) [22]. Akahoshi et al. studied 53 subepithelial gastric tumors. Diagnostic specimens were obtained in 42/51 (82%) patients [17]. In this study, the collection rate of adequate specimens from a gastric subepithelial hypoechoic tumor with continuity to proper muscle layer was 74.5%. No major complications were encountered. A reason for lower diagnostic yield in our study is that we defined, as diagnostic, EUS-FNA procedure only in a case of gaining a sufficient material for immunohistological diagnosis, which is essential for a diagnosis of GIST [1].

The predictive factors for inadequate EUS-FNA in gastric SMT were age and location in the stomach. Against our expectations, in younger patients, the rate of achieving adequate quantity of specimens was lower than that in older patients. The numbers of larger SMT in younger and older patients were 7/20 (35.0%) and 15/27 (55.6%), respectively, which showed no significant difference (*P* = .16, chi-square test). We speculated that it might be caused by the difficulty to keep the good position and view in EUS-FNA which could be easily influenced by a stronger pharyngeal reflex of young patients compared to older patients. The location of SMT in the stomach was shown in Figure 1. In fact, SMTs at L area were difficult to obtain adequate samples. We think it was more difficult to keep a scope stably at the L area, because we need to keep pushing a scope only at the L area, and the situation is unstable.

This study has several important strengths. This is one of a few studies about the factors that influence the diagnostic yield of EUS-FNA for SMT. Other study reports that the sensitivity of EUS-FNA is influenced by size, organ, shape, and layer of origin [25]. 

But the report is not limited to gastric SMTs, and the number is lesser in this study.

Additionally, because there were no study about the factors associated with inadequate tissue yield, this is precious, especially in the point of the analysis of each location in a stomach.

 In conclusion, the diagnostic yield of EUS-FNA for the diagnosis of gastric SMT is 74.5%, and the factors associated with inadequate tissue yield in EUS-FNA for gastric SMT are younger age and location of lesion at L area in a stomach. Considering these factors may bring the improvement of accuracy in EUS-FNA. 

## Figures and Tables

**Table 1:** Technical results of EUS-FNA (*n* = 47).

	Results
Gaining an adequate specimen for immunohistological diagnosis	35/47 (74.5%)
Complications (Bleeding)	0/47 (0%)
Accuracy*	13/13 (100%)

*Compared to the pathological findings using the specimens by surgery.

**Table 2:** Factors associated with the diagnostic yield of EUS-FNA.

	All	Diagnostic	Nondiagnostic	
Factors	(*n* = 47)	(*n* = 35)	(*n* = 12)	*P* value
Age (years old)	60.4 ± 13.3	64.4 ± 11.3	49.0 ± 12.3	<.001*
20*∼*29	1 (2.1%)	0 (0%)	1 (8.3%)	
30*∼*39	3 (6.4%)	1 (2.9%)	2 (16.7%)	
40*∼*49	5 (10.6%)	2 (5.7%)	3 (25.0%)	
50*∼*59	11 (23.4%)	8 (22.9%)	3 (25.0%)	
60*∼*69	17 (36.2%)	14 (40.0%)	3 (25.0%)	
70*∼*79	6 (12.8%)	6 (17.1%)	0 (0%)	
80*∼*89	4 (8.5%)	4 (11.4%)	0 (0%)	
Sex (M/F)	24/23	19/16	5/7	N.S.**
Tumor location				
U	26 (55.3%)	22 (62.9%)	4 (33.3%)	N.S.**
M	15 (31.9%)	11 (31.4%)	4 (33.3%)	N.S.**
L	6 (12.8%)	2 (5.7%)	4 (33.3%)	.013 **
Tumor size (mm)	29.0 ± 11.2	29.3 ± 12.4	28.3 ± 6.7	N.S.*
10*∼*19	5 (10.6%)	4 (11.4%)	1 (8.3%)	
20*∼*29	20 (42.6%)	14 (40%)	5 (41.7%)	
30*∼*39	14 (29.8%)	9 (25.7%)	5 (41.7%)	
40*∼*49	7 (14.9%)	6 (17.1%)	1 (8.3%)	
50*∼*	1 ( 2.1%)	1 (2.9%)	0 (0%)	
Pattern of growth (No.[%])				N.S.**
intragastric	34 (72.4%)	23 (65.7%)	11 (32.4%)	
extragastric	5 (10.6%)	5 (14.3%)	0 (0%)	
mixed	8 (17.0%)	7 (20.0%)	1 (12.5%)	
Pattern of echography (No.[%])				N.S.**
homo	20 (42.6%)	13 (37.1%)	7 (35.0%)	
hetero	27 (57.4%)	22 (62.9%)	5 (18.5%)	

*Student *t*-test.

**Chi-square test.

**Table 3:** Logistic regression analysis of factors associated with nondiagnostic yield of EUS-FNA.

	Univariate Analysis		Multivariate Analysis	
	Hazard Ratio (95% confidence interval)	*P* value	Hazard Ratio (95% confidence interval)	*P* value
Sex (Male)	0.564 (0.149–2.137)	.564		
Age (<60 years)	9.167 (1.720–48.85)	.009	11.91 (1.761–80.48)	.011
Location (Lower)	8.000 (1.238–51.69)	.029	10.62 (1.290–87.42)	.028
Tumor Size (>29 mm)	0.564 (0.149–2.137)	.564		
Pattern of growth (Intragastric)	2.956 (0.732–11.93)	.128		
Pattern of echography (Homo)	5.739 (0.660–49.91)	.113		

The thresholds of age and tumor size were their average values.

## Abbreviations

EUS-FNA: Endoscopic ultrasound-guided fine-needle aspiration biopsy
SMT: Submucosal tumor
GIST: Gastrointestinal stromal tumor
CI: Confidence interval.
